# NiCo_2_S_4_ Nanocrystals on Nitrogen-Doped Carbon Nanotubes as High-Performance Anode for Lithium-Ion Batteries

**DOI:** 10.1186/s11671-021-03562-7

**Published:** 2021-06-12

**Authors:** Haisheng Han, Yanli Song, Yongguang Zhang, Gulnur Kalimuldina, Zhumabay Bakenov

**Affiliations:** 1 School of Materials Science and Engineering, Tianjin Key Laboratory of Materials Laminating Fabrication and Interface Control Technology, Hebei University of Technology, Tianjin, 300130 China; 2 Department of Mechanical and Aerospace Engineering, Nazarbayev University, Nur-Sultan, 010000 Kazakhstan; 3 Department of Chemical and Materials Engineering, National Laboratory Astana, Nazarbayev University, Nur-Sultan, 010000 Kazakhstan

**Keywords:** Anode, NiCo_2_S_4_, Nitrogen-doped carbon nanotube, Lithium-ion batteries, Binary metal sulfides

## Abstract

In recent years, the development of lithium-ion batteries (LIBs) with high energy density has become one of the important research directions to fulfill the needs of electric vehicles and smart grid technologies. Nowadays, traditional LIBs have reached their limits in terms of capacity, cycle life, and stability, necessitating their further improvement and development of alternative materials with remarkably enhanced properties. A nitrogen-containing carbon nanotube (N-CNT) host for bimetallic sulfide (NiCo_2_S_4_) is proposed in this study as an anode with attractive electrochemical performance for LIBs. The prepared NiCo_2_S_4_/N-CNT nanocomposite exhibited improved cycling stability, rate performance, and an excellent reversible capacity of 623.0 mAh g^–1^ after 100 cycles at 0.1 A g^–1^ and maintained a high capacity and cycling stability at 0.5 A g^–1^. The excellent electrochemical performance of the composite can be attributed to the unique porous structure, which can effectively enhance the diffusivity of Li ions while mitigating the volume expansion during the charge–discharge processes.

## Background

Lithium-ion battery (LIB) is a leading battery technology used in portable electronic devices, electric vehicles, and renewable energy storage [1, 2]. Therefore, the development of LIBs with a high energy density has become a research direction crucial for the sustainable development of various sectors of economics and industry [3–5]. For instance, the specific energy density of a commercial graphite anode material reached its theoretical capacity of 372 mAh g^–1^, which does not leave much room for its further enhancement to satisfy the performance requirements of emerging electronics and electric vehicle technologies [6, 7]. As a result, it is essential to develop alternative anode materials for LIBs to satisfy the needs of the modern society.

Transition-metal sulfides (TMSs) offer remarkably higher specific capacity than traditional electrode materials [8–12]. Recently, TMSs have been reported as anodes with excellent conductivity and catalytic activity. Among them, binary nickel–cobalt sulfide (NiCo_2_S_4_) exhibits a high theoretical specific capacity (703 mAh g^–1^), an excellent electronic conductivity (1.26 × 10^6^ S m^–1^), and a greater abundance of redox reaction sites [13–17]. The reported general charge/discharge mechanism of NiCo_2_S_4_ with lithium (Li) involves the following reactions:

1 $${\text{NiCo}}_{2} {\text{S}}_{4} + 8{\text{Li}}^{ + } + 8{\text{e}}^{{-}} \to {\text{Ni}} + {\text{Co}} + 4{\text{Li}}_{2} {\text{S}}$$

2 $${\text{Ni}} + x{\text{Li}}_{2} {\text{S}} \leftrightarrow {\text{NiS}}_{x} + 2x{\text{Li}}^{ + } + 2x{\text{e}}^{{-}}$$

3 $${\text{Co}} + x{\text{Li}}_{2} {\text{S}} \leftrightarrow {\text{CoS}}_{x} + 2x{\text{Li}}^{ + } + 2x{\text{e}}^{{-}}$$

However, despite the large Li storage capacity of NiCo_2_S_4_, there are still problems related to the low reversibility of charge/discharge processes due to the accompanying volume variation, leading to material disintegration and consequently severe capacity fading [18]. Another serious problem originates from the shuttle effect of polysulfides produced by the dissolution of lithium polysulfide (LPS) in the electrolyte, resulting in a low capacity retention as well [19, 20].

To overcome the issues of NiCo_2_S_4_ anodes related to the volume change and LPS dissolution, various approaches including nanostructuring and use of carbonaceous additives and carbon-based hosts have been developed with promising results. Nanostructuring and its combination with carbon/graphene networks can increase the electrode–electrolyte interface contact area and shorten the Li-ion pathways, leading to a higher specific capacity [18]. Therefore, this study reports the in situ growth of NiCo_2_S_4_ nanoparticles onto carbon nanotubes (CNT) structure using a hydrothermal method. Furthermore, to increase the electroactivity of the electrode material, the nitrogen (N) heteroatoms were incorporated into the CNT matrix. Such a processing makes N-CNT more conducive, leading to the uniform growth of NiCo_2_S_4_ and thus improving the crystallinity of NiCo_2_S_4_/N-CNT anode. In this unique structure, CNT forms an elastic matrix that enhances the structural stability, improves the ionic conductivity of the composite, and mitigates the volume variation of NiCo_2_S_4_ particles. The NiCo_2_S_4_/N-CNT material maintains good capacity retention during cycling and significantly restrains the voltage fading. The NiCo_2_S_4_/N-CNT composite anode exhibits an initial discharge capacity of 1412.1 mAh g^–1^ at 0.1 A g^–1^, and the discharge capacity remains at 623.0 mAh g^–1^ after 100 cycles.

## Methods

### ***Synthesis of NiCo***_***2***_***S***_***4***_

First, 0.074 g of Co(AC)_2_·4H_2_O and 0.037 g of Ni(Ac)_2_·4H_2_O were dissolved in 40 mL ethanol. The solution was stirred on a water bath at 80 °C for 2 h and at room temperature for another 2 h. Then, 0.078 g of thiourea was added to the mixture, which was further continuously stirred for 20 h before transferring the reaction mixture to a 100 mL autoclave. The hydrothermal reaction was carried out at 170 °C for 3 h. After cooling to room temperature, the product was washed several times with deionized water and freeze-dried under reduced pressure.

### ***Synthesis of NiCo***_***2***_***S***_***4***_***/N-CNT Nanocomposites***

First, 68 mg of mildly oxidized CNT was ultrasonically dispersed in 40 mL of ethanol. Then, 0.074 g of Co(AC)_2_·4H_2_O and 0.037 g of Ni(Ac)_2_·4H_2_O were added, and the mixture was stirred on a water bath at 80 °C for 2 h. Next, 2 mL of NH_3_·H_2_O and 0.078 g of thiourea were added to the solution, and the reaction mixture was stirred for 2 h. The reaction mixture was transferred to a 50-mL autoclave, followed by a hydrothermal reaction at 170 °C for 3 h. The product was cooled to room temperature and centrifuged with deionized water several times and freeze-dried. NiCo_2_S_4_/CNT was synthesized following the same method but without the addition of NH_3_·H_2_O.

### Characterization of Materials

The crystal structure of the as-synthesized samples was characterized by powder X-ray diffraction (XRD, D8 Discover Bruker). X-ray photoelectron spectrometry (XPS) was performed to analyze the elemental composition of the samples using a K-Alpha 1063 analyzer. The morphology of the samples was studied using a scanning electron microscope (SEM, JSM-7100F, JEOL) and a transmission electron microscope (TEM, JEM-2100F). The specific surface area of the samples was calculated using the Brunauer–Emmett–Teller (BET) method based on the N_2_ adsorption–desorption isotherms obtained by using a V-Sorb 2800P. Thermogravimetric analysis (TGA) was carried out in air with a heating rate of 10 °C min^−1^.

### Electrochemical Measurements

The electrochemical performance of NiCo_2_S_4_/N-CNT samples was evaluated in CR 2032 coin-type cells. To prepare the electrode slurry, 70 wt% of NiCo_2_S_4_/N-CNT composite, 15 wt% of carbon black (Super P), and 15 wt% of polyvinylidene fluoride (PVDF) binder were mixed in 1-methyl-2-pyrrolidinone (NMP). The slurry was uniformly spread onto a Cu foil using a doctor blade technique and then dried at 70 °C for 8 h in air. Circular disk electrodes were cut after drying, and the cells were assembled in a high-purity Ar-gas (99.9995%) filled glove box (MBraun). The mass loading of NiCo_2_S_4_/N-CNT in the electrodes was about 2 mg cm^–2^. Pure Li foils were used as reference and counter electrodes, and microporous polypropylene Celgard 2300 was used as a separator. The electrolyte was 1 mol L^–1^ LiPF_6_ (Aladdin, CAS number: 21324-40-3) in a mixture of ethylene carbonate (EC, Aladdin, CAS number: 96-49-1) and dimethyl carbonate (DMC, CAS number: 616-38-6) with a volume ratio of 1:1. The galvanostatic charge/discharge measurements were conducted using a multichannel battery testing system (Neware BTS4000) at a potential window of 0.01–3.00 V (vs. Li^+^/Li). Cyclic voltammetry (CV) and electrochemical impedance spectroscopy (EIS) were performed using an electrochemical workstation (Princeton, VersaState4).

## Results and Discussion

**Scheme 1:**
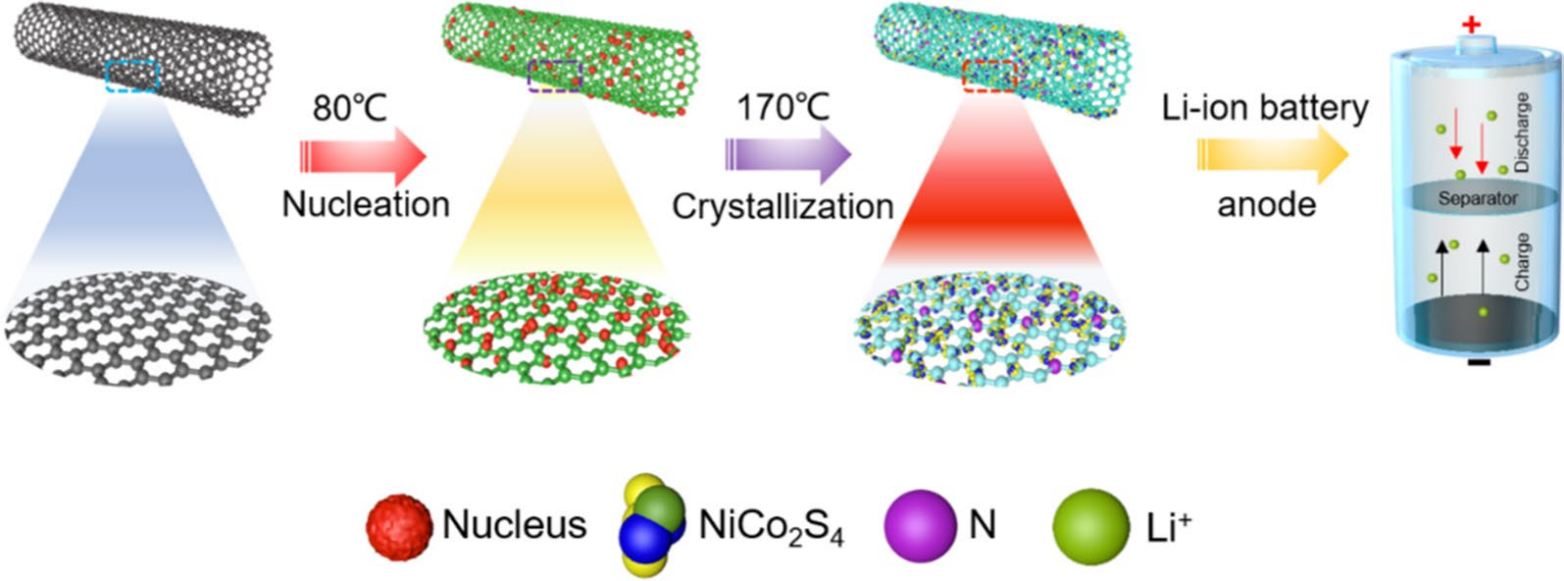
Schematic representation of NiCo_2_S_4_/N-CNT composite

**Fig. 1:**
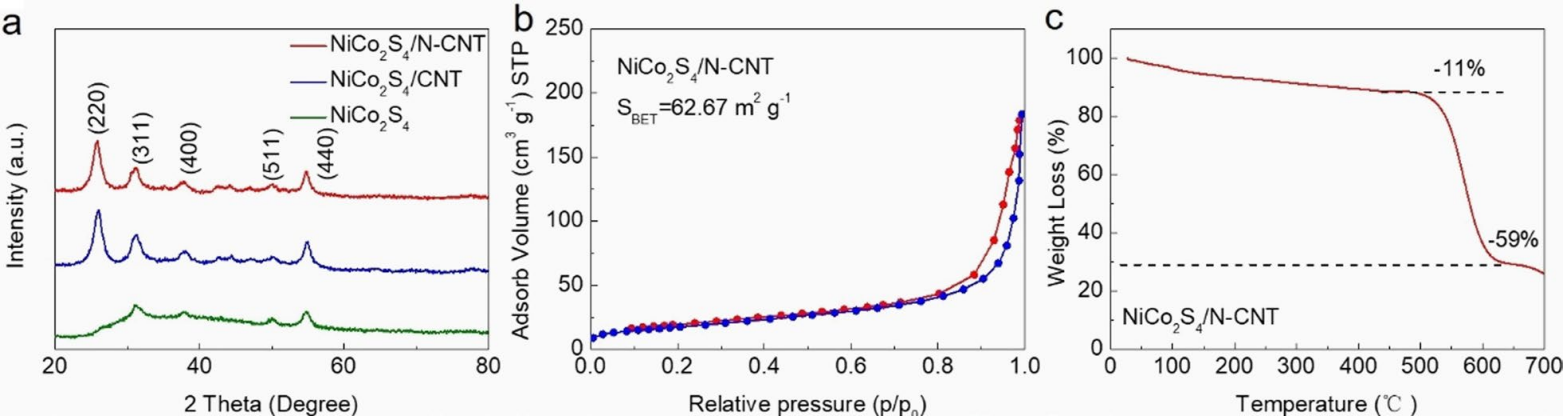
**a** XRD patterns of NiCo_2_S_4_, NiCo_2_S_4_/CNT, and NiCo_2_S_4_/N-CNT; **b** N_2_ adsorption–desorption isotherms of NiCo_2_S_4_/N-CNT; **c** TGA of NiCo_2_S_4_/N-CNT

Scheme 1 shows the preparation route of NiCo_2_S_4_/N-CNT composite. Initially, the surface of CNT was pretreated with a solution of Ni^2+^ and Co^2+^. Then, the N atoms were doped into the CNTs via a hydrothermal reaction at 170 °C, while NiCo_2_S_4_ was grown in situ on the surface of CNTs. The crystal structures of NiCo_2_S_4_, NiCo_2_S_4_/CNT, and NiCo_2_S_4_/N-CNT composites were characterized by XRD (Fig. 1a). The characteristic diffraction peaks of NiCo_2_S_4_ (JCPDS 20-0728) were observed in all the three samples. Moreover, the peaks in NiCo_2_S_4_/N-CNT were more pronounced and sharper than those in NiCo_2_S_4_/CNT [21]. It is believed that N-CNT can be used as active nucleation sites to promote the uniform and dense growth of NiCo_2_S_4_ [22]. Figure 1b shows the BET results for the NiCo_2_S_4_/N-CNT nanocomposites. The specific surface area of NiCo_2_S_4_/N-CNT nanocomposites is 62.67 m^2^ g^−1^. As shown in the TGA analysis data (Fig. 1c), the NiCo_2_S_4_/N-CNT nanocomposite exhibited a weight loss at a temperature range of 400–600 °C, which was caused by the combustion of CNTs. Therefore, the content of NiCo_2_S_4_ in the NiCo_2_S_4_/N-CNT composite was determined as ~ 30 wt%.

**Fig. 2:**
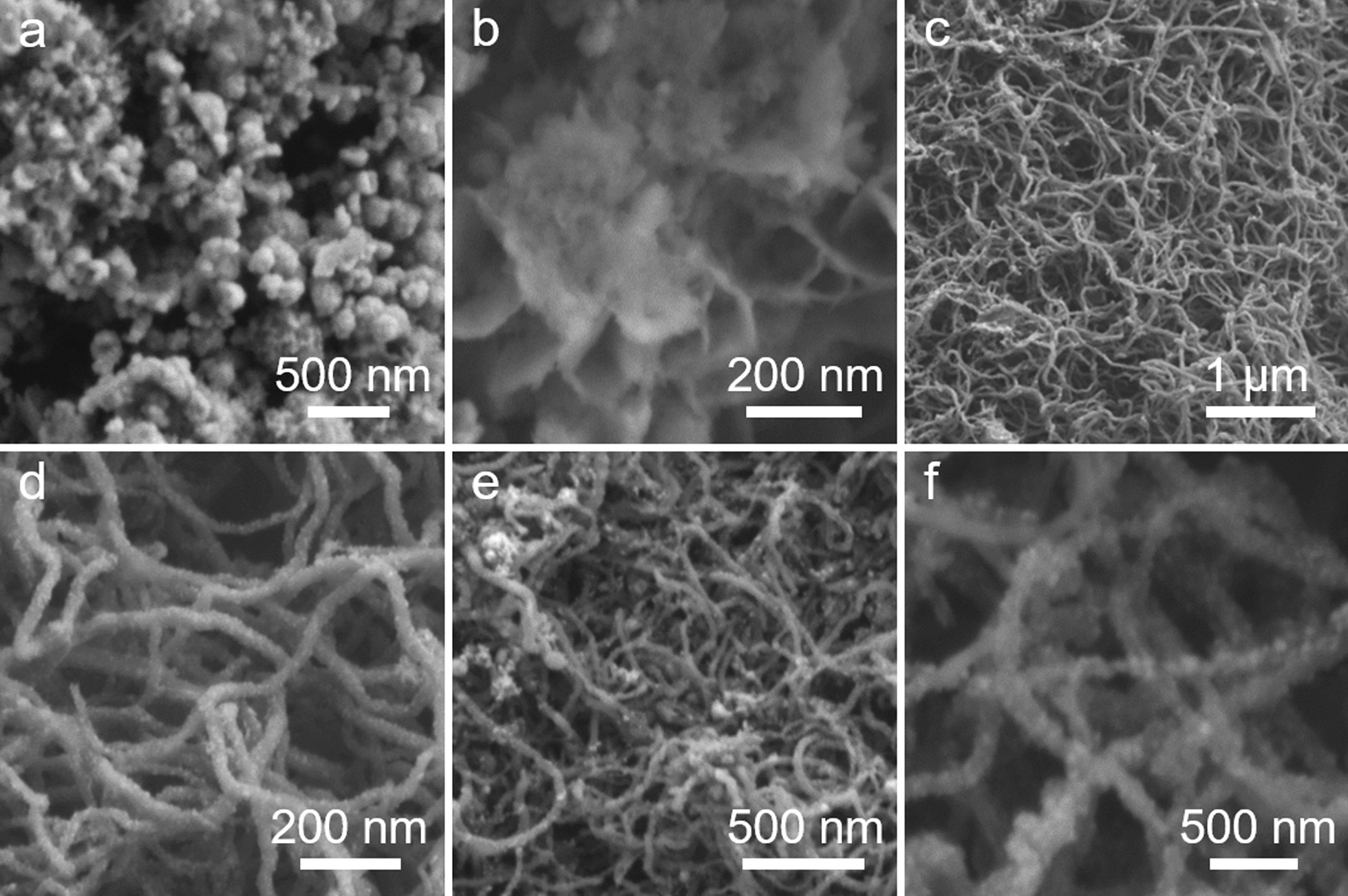
SEM images of **a**, **b** NiCo_2_S_4_; **c**, **d** NiCo_2_S_4_/CNT; and **e**, **f** NiCo_2_S_4_/N-CNT

The SEM results of the samples are shown in Fig. 2a, b. The as-synthesized NiCo_2_S_4_ nanoparticles appear to be more tightly packed and agglomerated. On the other hand, through the introduction of CNT and N-CNT, the NiCo_2_S_4_ nanoparticles were uniformly distributed and deposited to form NiCo_2_S_4_/CNT composite (Fig. 2c, d) and NiCo_2_S_4_/N-CNT (Fig. 2e, f), respectively. However, the density of NiCo_2_S_4_ nanoparticles on the N-CNT surface in NiCo_2_S_4_/N-CNT was significantly higher than that in the NiCo_2_S_4_/CNT composite. This confirms that the introduction of N atoms in CNTs promotes the denser growth of NiCo_2_S_4_ nanoparticles.

**Fig. 3:**
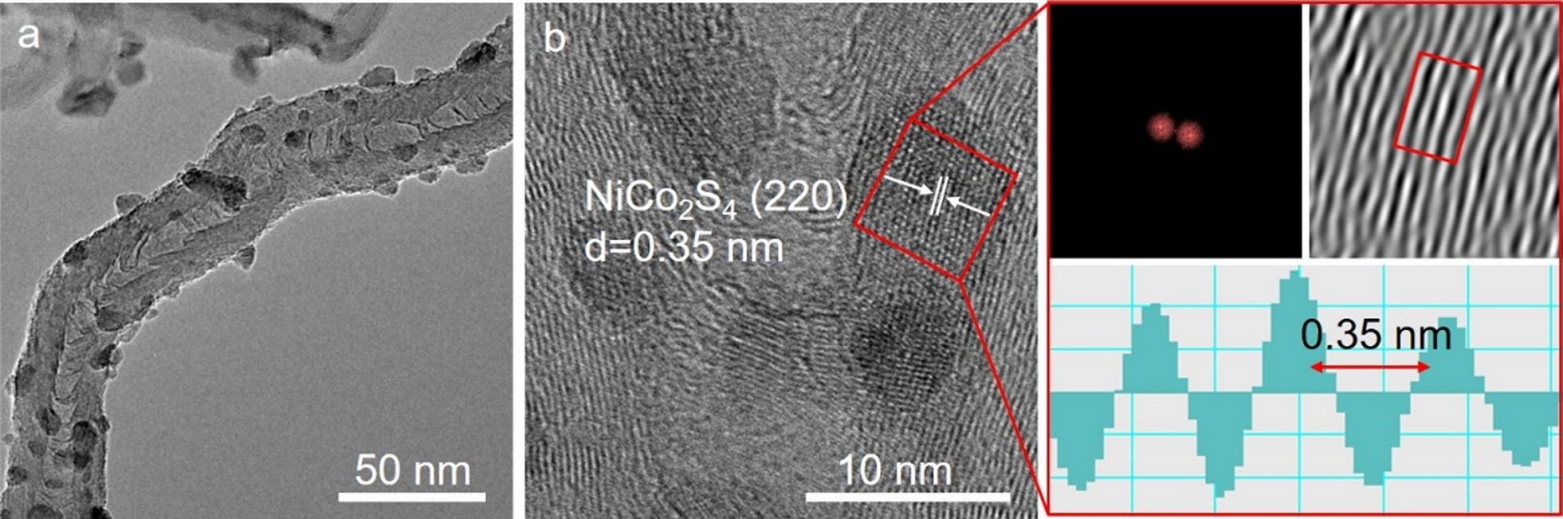
**a** TEM image; **b** HRTEM image of NiCo_2_S_4_/N-CNT

The TEM images in Fig. 3a show that the NiCo_2_S_4_ particles have an average diameter of ~ 5 nm and are uniformly distributed on the surface of N-CNTs. In the high-resolution TEM (HRTEM) image of NiCo_2_S_4_/N-CNT shown in Fig. 3b, the nanoparticles of about 5 nm in diameter exhibit a clear lattice fringe of 0.35 nm, corresponding to the (220) plane of NiCo_2_S_4_. Besides, many crooked graphitic lattice fringes were observed around the nanoparticles. The fast Fourier transform (FFT) and lattice spacing profiles in Fig. 3b further confirmed the incorporation of NiCo_2_S_4_ nanoparticles into the N-CNT structure.

**Fig. 4:**
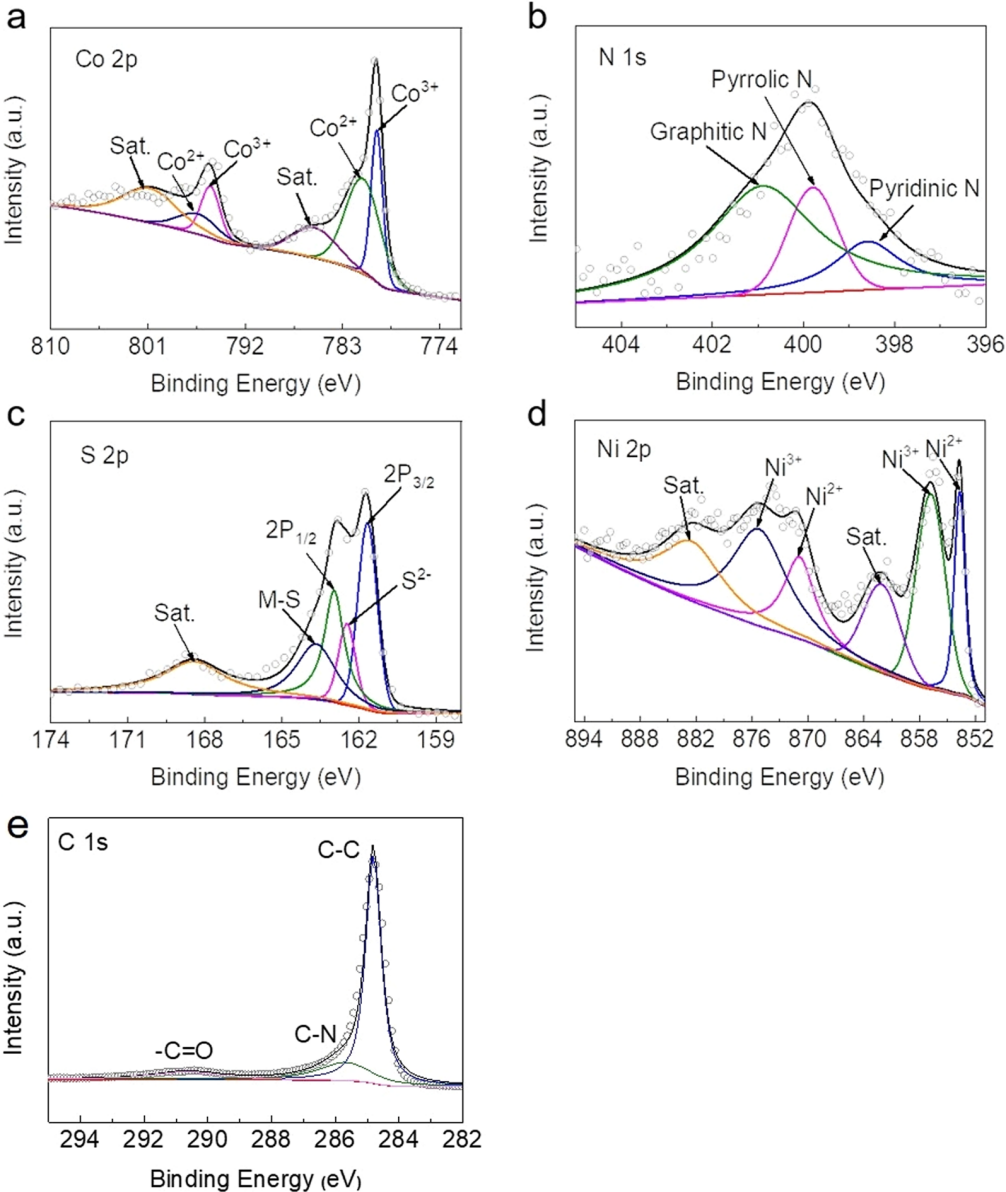
XPS spectra of **a** Co 2*p*, **b** N 1*s*, **c** S 2*p*, **d** Ni 2*p*, and **e** C 1*s* in NiCo_2_S_4_/N-CNT

Further, XPS was used to determine the bonding characteristics and surface chemical composition of NiCo_2_S_4_/N-CNT. The Co 2*p* spectra (Fig. 4a) can be divided into two peaks at 778.8 eV and 793.0 eV, corresponding to Co^3+^ and Co^2+^, respectively [23, 24]. In the N 1*s* spectrum (Fig. 4b), the peaks at 398.3, 399.7, and 400.9 eV can be assigned to the pyridinic, pyrrolic, and graphitic N, respectively [25, 26]. In the XPS spectrum of S 2*p* (Fig. 4c), the S 2*p*_3/2_ and S 2*p*_1/2_ can be clearly observed at 161.2 and 163.1 eV, respectively, and the peak at 163.8 eV corresponds to the metal-sulfur bond [27, 28]. As shown in Fig. 4d, in addition to the satellite peaks, the binding energies of Ni 2*p* centered at 854.6 and 856.9 eV correspond to Ni 2*p*_3/2_, and those at 871.1 and 875.5 eV correspond to Ni 2*p*_1/2_. This indicates the presence of both Ni^3+^ and Ni^2+^ in the sample [29, 30]. As shown in Fig. 4e, three fitting peaks are present in the C1s profile at 284.9, 285.7, and 290.4 eV, which can be attributed to C–C, C–N, and –C=O bonds, respectively. In summary, the XPS of NiCo_2_S_4_/N-CNT indicated the formation of a highly ordered crystal structure of NiCo_2_S_4_ and demonstrated the successful introduction of N element into the structure of compounds.

**Fig. 5:**
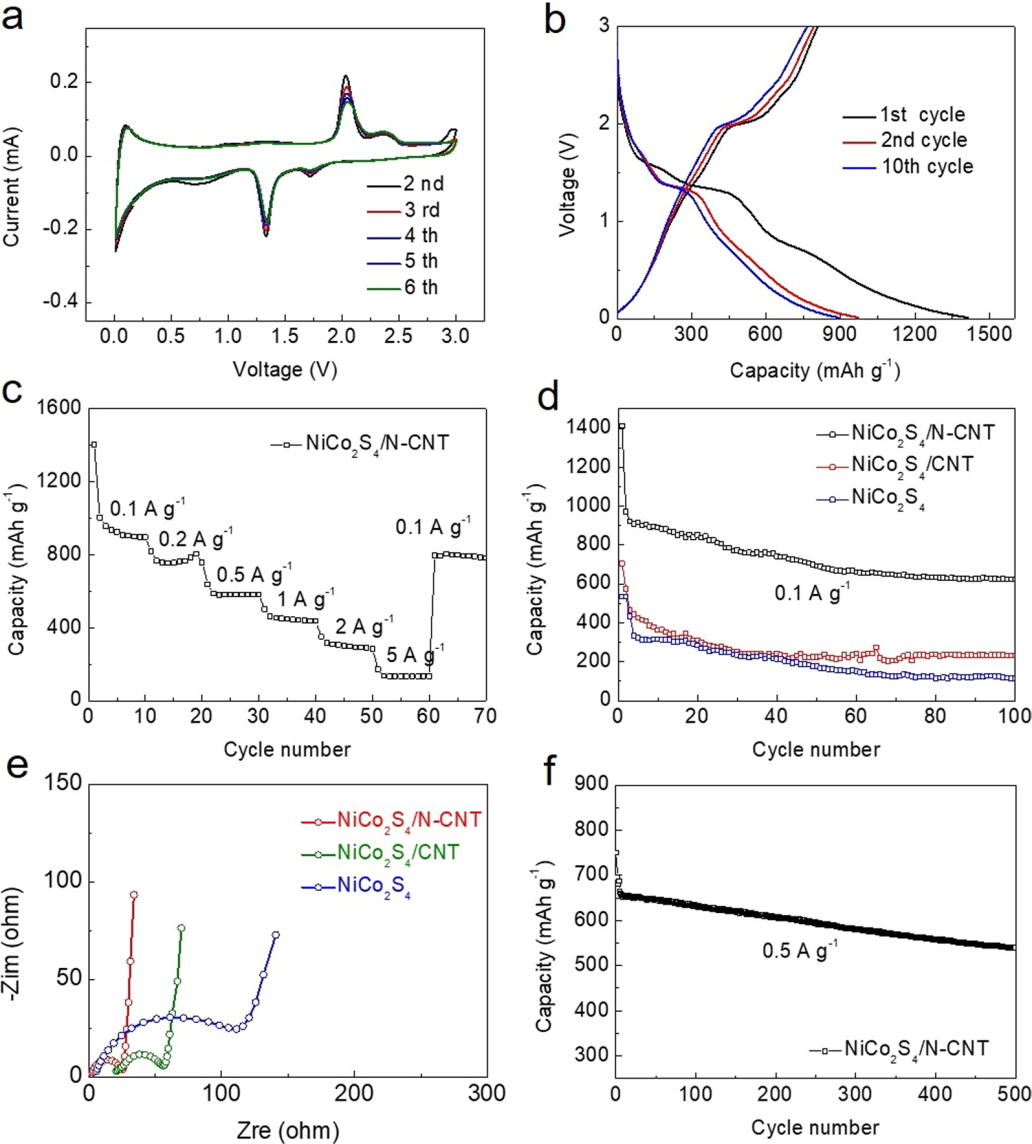
**a** CVs of NiCo_2_S_4_/N-CNT at a scan rate of 0.1 mV s^−1^ between 0.01 and 3.0 V versus Li^+^/Li; **b** charge/discharge curves of NiCo_2_S_4_/N-CNT at 0.1 A g^−1^; **c** rate capability of NiCo_2_S_4_/N-CNT electrodes at various current rates; **d** cycling performance of NiCo_2_S_4_/N-CNT, NiCo_2_S_4_/CNT, and NiCo_2_S_4_ at 0.1 A g^–1^; **e** EIS plots of NiCo_2_S_4_/N-CNT, NiCo_2_S_4_/CNT, and NiCo_2_S_4_; **f** cycling performance at a current density of 0.5 A g^−1^

The electrochemical characteristics of NiCo_2_S_4_/N-CNT for Li storage were evaluated by CV and charge–discharge cycling, as shown in Fig. 5 at a potential range of 0.01–3.00 V (vs. Li^+^/Li). The cathodic process consisted of three reduction peaks (Fig. 5a) situated at 1.71 V, 1.33 V, and 0.70 V. The strongest peak is positioned at 1.33 V, and two weaker peaks correspond to the reduction of NiCo_2_S_4_ to Ni and Co. In comparison, the peaks at 1.71 V and 0.70 V correspond to the formation of Li_2_S and the SEI film, respectively. In the anodic process, the oxidation peaks at 1.33 V and 2.05 V can be attributed to the oxidation of metallic Co to CoS_x_. In addition, there is an intensive peak at 2.32 V resulting from the oxidation reactions of metallic Ni and Co to NiS_x_ and CoS_x_, respectively. The shape of the curve, peak position, and the intensity of peaks are relatively stable in the following cycles, indicating that NiCo_2_S_4_/N-CNT has good stability and reversibility.

Figure 5b shows the charge–discharge curves of NiCo_2_S_4_/N-CNT at 0.1 A g^–1^ for the 1st, 2nd, and 10th cycles. The first charge and discharge capacities of the NiCo_2_S_4_/N-CNT electrode reached 807.6 and 1412.1 mAh g^–1^, respectively, with the initial coulombic efficiency of 57.2%. The discharge capacities of the 2nd and 10th cycles are 970.7 mAh g^–1^ and 891.1 mAh g^–1^, respectively. The reversibility of the charge/discharge process improved with the cycle number accompanied with an increased coulombic efficiency. The obtained CV profiles correspond to the charge/discharge curves of NiCo_2_S_4_/N-CNT.

To further study the electrochemical performance of NiCo_2_S_4_/N-CNT, the rate capability was evaluated at current densities from 0.1 to 5 A g^–1^ (Fig. 5c). The results indicate that the capacity of NiCo_2_S_4_/N-CNT decreased with the increase in current density. When the current density was returned to 0.1 A g^−1^, the capacity of NiCo_2_S_4_/N-CNT returned to a value of 796.1 mAh g^–1^, exhibiting about 84% capacity retention and demonstrating that NiCo_2_S_4_/N-CNT exhibits an excellent rate performance. The cycling performance data of NiCo_2_S_4_/N-CNT, NiCo_2_S_4_/CNT, and NiCo_2_S_4_ for 100 cycles at 0.1 A g^–1^ are shown in Fig. 5d. For the initial 50 cycles, the anode undergoes a slight capacity fading. Then, NiCo_2_S_4_/N-CNT anode stabilized its capacity for the rest of the cycles and demonstrated a value of 623.0 mAh g^–1^ after 100 cycles. These results show that compared with the NiCo_2_S_4_/CNT and NiCo_2_S_4_ electrodes, the NiCo_2_S_4_/N-CNT electrode exhibited a remarkably higher discharge specific capacity and better cycle stability. Figure 5e shows the EIS data. The high-frequency semicircles in the Nyquist plots correspond to the charge transfer resistance (*R*_ct_) of the electrodes. The NiCo_2_S_4_/N-CNT electrode clearly exhibits the lowest *R*_ct_ values, suggesting a remarkably enhanced charge/mass transfer kinetics. Figure 5f shows the cycling performance of NiCo_2_S_4_/N-CNT electrode at 0.5 A g^−1^ over 500 cycles. The NiCo_2_S_4_/N-CNT electrode delivers an initial specific discharge capacity of 750.2 mAh g^−1^ and maintains a reversible capacity of 539.3 mAh g^−1^ after 500 cycles, further confirming an excellent cycling and rate capability of this high capacity anode for lithium batteries.

## Conclusions

In summary, a NiCo_2_S_4_/N-CNT composite was prepared using a one-pot facile hydrothermal synthesis route. By introducing the N atoms into the CNT structure, uniformly distributed NiCo_2_S_4_ nanoparticles with reduced particle sizes were obtained. The assembled cells with the NiCo_2_S_4_/N-CNT anode demonstrated a high specific capacity of about 623.0 mAh g^–1^ and excellent cycling stability at 0.1 A g^–1^ after 100 cycles. Furthermore, this electrode exhibited an excellent cycling property at 0.5 A g^−1^ over 500 cycles, confirming its ability to maintain its high performance at elevated current densities. Our study shows that this synthesis method is a feasible way to grow NiCo_2_S_4_ nanoparticles with uniform distribution on the surface of a CNT substrate as a high-performance anode for LIBs.

## Data Availability

All data generated or analyzed during this study are included in this published article.

## Abbreviations

LIBs: Lithium-ion batteries
CNT: Carbon nanotube
NiCo_2_S_4_: Binary nickel–cobalt sulfide
PVDF: Polyvinylidene fluoride
NMP: *N*-Methyl-2-pyrrolidone
XRD: X-ray powder diffraction
SEM: Scanning electron microscopy
CV: Cyclic voltammetry
EIS: Electrochemical impedance spectroscopy
N-CNT: Nitrogen-containing carbon nanotube
TMS: Transition metal sulfides
LPS: Lithium polysulfide
XPS: X-ray photoelectron spectrometry
TEM: Transmission electron microscope
HRTEM: High-resolution transmission electron microscope
BET: Brunauer–Emmett–Teller
TGA: Thermogravimetric analysis
EC: Ethylene carbonate
DMC: Dimethyl carbonate
FFT: Fast Fourier transform
N: Nitrogen
Li: Lithium
